# Energy Economics and Policy

**DOI:** 10.1155/2013/536517

**Published:** 2013-05-09

**Authors:** Zhan-Ming Chen, Bin Chen, Han-Song Tang

**Affiliations:** ^1^School of Economics, Renmin University of China, Beijing 100872, China; ^2^School of Environment, Beijing Normal University, Beijing 100875, China; ^3^Department of Civil Engineering, City College, The City University of New York, New York, NY 10031, USA

There are emerging concerns worldwide on management of energy resources and its development, such as control of the environmental impact of energy use, regulation and expedition of the commercialization of renewable energy, and reevaluation of the safety issue of nuclear power. As a result, theoretical and empirical studies on energy economics and relevant policies are of emergent need to assess the performance of previous efforts as well as to guide future development. Especially, investigations on the characters of energy market and governments' roles in it are of significant implication for decision makers concerning different aspects of energy management.

20 papers selected from 36 submissions are published in this issue, which cover many important topics in the field of energy economics and policy. At the macroscale, P. Massot and Z.-M. Chen review the history of global uranium market and discuss the potential coexistence between the rising market of China and the rest of the world. X. H. Xia and Y. Hu use China's subprovince and prefecture level data to analyze the determinants of electricity consumption intensity. B. Zhang et al. provide an overview of resources use and environmental impact of the Chinese industry during 1997–2006 based on an exergetic assessment. The fossil fuels inputs in China during 2000–2010 are estimated by S. Wang et al. in a material flow analysis which takes hidden flows into account. Acknowledging the importance of infrastructure investment for the Chinese economy, the embodied energy use in China's infrastructure investment during 1992–2007 is evaluated by H. Liu et al. based on an energy input-output model. S. Lee et al. analyze the potential economic and environmental effects of carbon tax in Japan using a global macroeconometric model. In the study by F. Tao et al., the directional distance function and the Luenberger productivity index are applied to measure the industry efficiency and total factor productivity at the level of subindustry in China during 1999–2009. By employing an input-output model, S. Guo et al. construct an embodied greenhouse gases emissions inventory for Beijing. Fuel consumption and exhaust emissions, including nitrogen oxides and particulate matters, by China's auto industry are estimated and related mitigation policies are discussed by Wu et al. In P. Dai et al.'s paper, an integrate optimization model is proposed for mitigating carbon dioxide emissions from the power sector of China.

Other efforts are devoted to energy systems at smaller scales. In the light of the concept of low-carbon community, S. Song et al. propose a life-cycle-based accounting framework for carbon dioxide emissions and employ it to a case in Beijing. As an efficient mode to organize modern production, the industrial park attracts special attentions not only for its important role in economic development but also for its highly concentrated energy use. Four papers in this issue discuss different aspects of the industrial park: the sustainability performance of the industrial park is evaluated based on a life-cycle multicriteria framework by J. Yang et al.; the carbon metabolism of the industrial park is simulated in an ecological network analysis by Y. Lu et al.; the greenhouse gas inventory and ecological-economic benefits of the industrial park are discussed in two papers by B. Chen and his colleagues. Another two papers in this issue focus on renewable energy systems: Y. Wang et al. compare the ecological-economic value of two different biogas plants by an emergy method; Q. Yang et al. assess the nonrenewable energy cost and greenhouse gas emissions of a “pig-biogas-fish” agricultural system based on a life-cycle assessment. To analyze the incentive mechanism of power plant efficiency retrofit, D. Yuan et al. calculate the internal rate of return of an ordinary steam turbine coal-fueled power plant in China under different scenarios. The price elasticity of residential electricity demand is estimated by G. Shi et al. to fill the gap between data requirement for policy making and scientific research outcome. In a more technical research, Z. Wang et al. focus on light extraction efficiency improvement of light-emitting diodes. 

In summary, economics analysis on energy systems and policies of their development is gaining more and more attention, and the papers presented in this special issue represent the current status of their research. Nevertheless, many problems remain unresolved, and more research is necessary. We look forward to new progress on the basis of and beyond the investigations reported in this issue.

*Zhan-Ming Chen* *Zhan-Ming Chen* *Bin Chen* *Bin Chen* *Han-Song Tang* *Han-Song Tang*

